# Supplementary material for: Analysis of Primary Chronic Lymphocytic Leukemia Cells’ Signaling Pathways
Source: Biomedicines. 2024 Feb 26;12(3):524. doi: 10.3390/biomedicines12030524 (PMC10968363; doi:10.3390/biomedicines12030524)
Supplement: Supplementary file 1 [file biomedicines-12-00524-s001.zip › biomedicines-2823051-supplementary.pdf]

biomedicines

MDPI

Supplementary Materials:

Supplementary Table S1. Clinical stage, age, sex and laboratory characteristics of patients (nos. 1-20) diagnosed with CLL according to the WHO classification. The patient score according to the Rai, Binet and TTM classification was reported. Samples for gene and protein analysis were taken at patients' first visit, before the start of treatment. TTM = total tumor mass. M = male, F = female,

CLL	AGE	SEX	LEUKOCYTES $(X\ 10^9/L)$	LYMPHOCYTES $(X\ 10^9/L)$	NEUTROPHILS $(X\ 10^9/L)$	HEMOGLOBIN (G/L)	THROMBOCYTES [illegible]	TTM	BINET	RAI
**1**	63	F	70	54	5	133	198	8	A	0
**2**	70	M	46	42	4	111	491	16	B	III
**3**	54	M	645	612	13	73	43	55	C	IV
**4**	66	F	239	227	7	67	171	20	C	III
**5**	69	F	77	59	5	96	85	14	C	IV
**6**	45	M	250	242	2	106	152	22	B	III
**7**	58	F	19	16	2	130	185	6	B	I
**8**	65	M	15	12	2	140	128	15	B	II
**9**	74	F	11	5	5	127	304	2	A	0
**10**	65	M	56	49	5	121	166	15	B	II
**11**	67	M	19	13	5	160	228	6	A	I
**12**	65	M	20	13	4	112	159	22	B	II
**13**	77	F	66	59	7	146	163	12	A	II
**14**	72	M	85	78	2	124	119	12	A	II
**15**	63	F	56	46	8	117	92	13	C	IV
**16**	50	F	64	59	4	138	217	9	A	I
**17**	75	F	107	99	5	133	183	9	A	I
**18**	66	M	23	20	1	92	98	6	C	IV
**19**	75	M	92	83	8	81	262	14	C	III
**20**	37	M	15	11	4	149	209	6	A	I

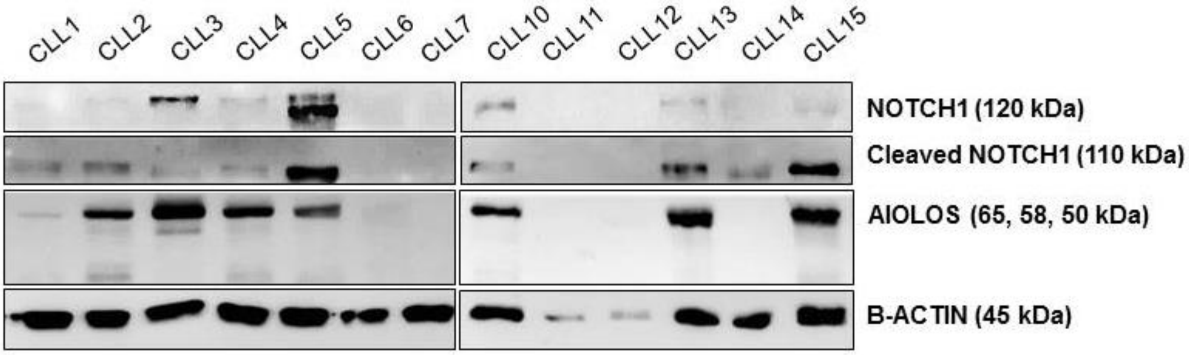

Supplementary Figure S1. Western blot analysis of B-CLL samples. Proteins were isolated from 13 CLL samples and analyzed for expression of NOTCH1, cleaved NOTCH1, AIOLOS and β-ACTIN using corresponding antibodies.
